# Individual Differences in Fornix Microstructure and Body Mass Index

**DOI:** 10.1371/journal.pone.0059849

**Published:** 2013-03-28

**Authors:** Claudia Metzler-Baddeley, Roland J. Baddeley, Derek K. Jones, John P. Aggleton, Michael J. O’Sullivan

**Affiliations:** 1 Cardiff University Brain Research Imaging Centre (CUBRIC), School of Psychology, and the Neuroscience and Mental Health Research Institute, Cardiff University, Cardiff, United Kingdom; 2 Department of Experimental Psychology, Bristol University, Bristol, United Kingdom; 3 Department of Clinical Neuroscience, Institute of Psychiatry, King’s College, London, United Kingdom; Bellvitge Biomedical Research Institute-IDIBELL, Spain

## Abstract

The prevalence of obesity and associated health conditions is increasing in the developed world. Obesity is related to atrophy and dysfunction of the hippocampus and hippocampal lesions may lead to increased appetite and weight gain. The hippocampus is connected *via* the fornix tract to the hypothalamus, orbitofrontal cortex, and the nucleus accumbens, all key structures for homeostatic and reward related control of food intake. The present study employed diffusion MRI tractography to investigate the relationship between microstructural properties of the fornix and variation in Body Mass Index (BMI), within normal and overweight ranges, in a group of community-dwelling older adults (53–93 years old). Larger BMI was associated with larger axial and mean diffusivity in the fornix (r = 0.64 and r = 0.55 respectively), relationships that were most pronounced in overweight individuals. Moreover, controlling for age, education, cognitive performance, blood pressure and global brain volume increased these correlations. Similar associations were not found in the parahippocampal cingulum, a comparison temporal association pathway. Thus, microstructural changes in fornix white matter were observed in older adults with increasing BMI levels from within normal to overweight ranges, so are not exclusively related to obesity. We propose that hippocampal-hypothalamic-prefrontal interactions, mediated by the fornix, contribute to the healthy functioning of networks involved in food intake control. The fornix, in turn, may display alterations in microstructure that reflect weight gain.

## Introduction

The prevalence of obesity and its associated health problems is growing in the developed world. To develop effective interventions for obesity prevention, it is important to understand the neural and psychological factors that contribute to chronic weight gain. In the current study we investigated in a group of community dwelling, high functioning older adults the relationship between individual differences in Body Mass Index (BMI) and variation in the white matter microstructure of the fornix, the principal fibre tract associated with hippocampal connections beyond the temporal lobe [1].

Research into hippocampal function has traditionally focused on learning and memory [2] but there is growing appreciation of its involvement in other functions [3] including the regulation of food intake. Hippocampal lesions in rats can result in increased appetitive behaviour and weight gain [4], [5]. Likewise, reduced hippocampal volume [6] and abnormal hippocampal activation in response to food stimulation or feeding manipulations [7] have been related to obesity, although the direction of causality remains unclear.

The hippocampus is known to be directly connected, *via* the fornix, with several regions involved in the control of food intake. One such target is the hypothalamus, a complex region involved in homeostatic functions [8]. Hippocampal projections reach the preoptic area, anterior hypothalamic nuclei, arcuate nucleus, ventromedial nucleus and lateral hypothalamic area [9]. The precommissural fornix also contains efferents to nucleus accumbens and the orbital frontal cortices [10], [11]. These connections innervate sites strongly implicated in the hedonic control of food intake [12], [13].

Diffusion MRI tractography [14], [15] was used to investigate the relationship between BMI and microstructure of the fornix, as well as a comparison tract, the parahippocampal cingulum, which also contains medial temporal lobe connections but is not directly linked to either medial prefrontal cortex or hypothalamus [16], [17]. Microstructural indices were derived for each tract. The fornix is a medium-sized pathway that runs through the ventricles and, hence, is particularly susceptible to cerebrospinal fluid (CSF) based partial volume artefacts in diffusion tensor imaging (DTI) indices [18]. This is especially problematic in older adults, as aging is associated with significant gray and white matter atrophy [19], [20]. To account for this confound, data were corrected with the Free Water Elimination (FWE) approach [21]. This method fits two tensors, an isotropic and an anisotropic compartment, to the diffusion data from each voxel and, hence, allows the elimination of CSF contamination in white matter microstructural indices.

Additional measurements were made of hippocampal and whole brain volume, and associations were controlled for potential confounds of volume measures, age, sex, education, cognitive function (verbal IQ and episodic memory), blood pressure and diabetes status.

## Methods

### Ethics Statement

This study was reviewed and approved by the South East Wales Research Ethics Committee and each participant provided informed, written consent. All investigations have been conducted according to the principles expressed in the Declaration of Helsinki.

### Participants

Forty-six participants over the age of 50 years were recruited for a project investigating aging and brain structure from the local community by advertisements posted on the internet, and in family physician waiting rooms, newsletters and mail shots. Participants were informed that the study was about aging and memory; BMI and risk factors were not mentioned explicitly so are unlikely to have influenced recruitment. Participants fulfilled the selection criteria for the study if they were free of a history of neurological and/or psychiatric disease, moderate or severe head injury, alcohol and/or drug abuse, memory or other cognitive decline, stroke, large artery or peripheral vascular disease, structural heart disease or heart failure, and contra-indications to MRI. Two participants withdrew from the study due to ill health and one participant was excluded due to a subsequent diagnosis of Parkinson’s Disease. After visual inspection of the structural MRI scans for overt pathology three further participants were excluded because of extensive white matter hyperintensities suggestive of cerebral microvascular disease [22]. One individual’s MRI data could not be analysed due to motion artefacts. Finally, one participant’s height was not recorded at the beginning of the study leaving 38 MRI and BMI datasets available for analysis in the present study (see Table 1).

**Table 1:** Participants’ background information.

*n*	38
Age	M = 67.9, SD = 8.6 (53–93 years)
Sex	22 female, 16 male
Handedness	36 right, 2 left
Body Mass Index	M = 24.9, SD = 3.5 (17.5–32.5)
Blood pressure systolic	M = 141.2, SD = 17.2 (106–186)
Blood pressure diastolic	M = 80.8, SD = 9.7 (65–109)
Number diabetic	1
Education	M = 15.6, SD = 3.01 (10–22 years)
National Adult Reading Test – IQ	M = 121, SD = 8 (90–131)
Verbal free recall	M = 32.9 (max 48), SD = 8.2 (14–48)
Geriatric Depression Scale 15	M = 1.21, SD = 1.6 (0–6)

Risk factors for cardiovascular disease such as increased Body Mass Index (BMI) (weight in kg/height in square meter), hypertension, diabetes mellitus, high levels of blood cholesterol, and smoking were documented. Each participant was weighed in their clothing without shoes on a mechanical scale. Standing height was measured using a tape measure placed in fixed position against the wall. Systolic and diastolic blood pressure and pulse were measured with a digital blood pressure monitor (Model UA-631; A&D Medical, Tokyo, Japan) whilst participants were relaxed and comfortably seated with their arm well supported on a table. Other cardiovascular risk factors were self-reported by participants in a comprehensive medical history questionnaire.

For 17 participants BMI fell within normal range (18.5–24.9), for 17 within overweight range (25–30), three participants were obese (BMI >30), and one individual was underweight (BMI <18.5). Sixteen participants reported medication to control hypertension, two reported statins to control cholesterol levels, and one person was on medication for Type II diabetes mellitus. Seventeen participants reported never to have smoked and twenty-one participants to have smoked in the past. The group performed at superior level of intelligence [Mean IQ = 121; SD = 8 in the National Adult Reading Test (NART)] [23] and within normal-superior range for their age in a number of memory and executive function tasks (Table 1 and for detailed description see [24]). In addition, participants were screened for depression with the 15 items Geriatric Depression Scale (GDS15/[25]. All participants scored within the normal range except for one individual whose score was on the conventional cut-off of 6.

### MRI Data Acquisition

Diffusion weighted MR data were acquired using a 3T GE HDx MRI system (General Electric Healthcare) with a twice-refocused spin-echo EPI sequence providing whole oblique axial (parallel to the commissural plane) brain coverage. Data acquisition was peripherally gated to the cardiac cycle. Data were acquired from 60 slices of 2.4 mm thickness, with a field of view of 23 cm, and an acquisition matrix of 96×96. TE was 87 ms and parallel imaging (ASSET factor = 2) was employed. The b-value was 1200 s/mm^−2^. In each imaging session, data were acquired with diffusion encoded along 30 isotropically distributed directions [26] and three non-diffusion weighted scans using an optimised gradient vector scheme. The acquisition time was approximately 13 minutes.

### Diffusion MRI Pre-processing

The acquired images were corrected for distortions, introduced by the diffusion-weighting gradients, and for motion artefacts using *ExploreDTI* [27]. This was achieved using a global affine registration of each image volume to the first non-diffusion weighted volume, using normalized mutual information as the cost-function, followed by appropriate re-orientation of the encoding vectors and modulation of the signal intensity by the Jacobian determinant of the transformation [28]. Two separate processing steps were then taken: the first was to fit a two compartment model using the FWE approach to the data, to yield partial volume corrected indices of fractional anisotropy (FA), mean diffusivity (MD), radial diffusivity (RD) and axial diffusivity (AD) [21]; the second was to use constrained spherical deconvolution (CSD) to extract, in each voxel, the fibre orientation density function (fODF) with the spherical harmonics order no higher than lmax = 6 [29].

### Diffusion MRI Tractography

Deterministic tractography was performed using *ExploreDTI* [27], [30]. The tracking algorithm estimated the peak in the fODF at each seed-point and propagated in 0.5 mm steps along this axis. Peaks in the fODF were then estimated at the new location and the tracking moved a further 0.5 mm along the axis subtending the smallest angle to the current trajectory. In this way a pathway was traced through the data until the fODF fell below an arbitrary threshold (in this case 0.1) or the direction of the pathway changed through an angle greater than 60°. The procedure was then repeated by tracking in the opposite direction back to the initial seed-point to reconstruct the whole pathway passing through the seed-point. Three dimensional fibre reconstructions were made by: (i) initial tracking of pathways in the whole brain using every voxel as a seed-point from which to initiate the tracking algorithm; (ii) the subsequent application of waypoint region of interest (ROI) gates (“AND”, “OR”, “NOT” gates following Boolean logic) to isolate specific tracts from the whole brain tractography data. ROIs were manually drawn on a colour coded fibre orientation map [31] for each individual dataset as described in [24] using landmark techniques that have previously been shown to be highly reproducible [15], [32].

#### Fornix

**Figure 1:**
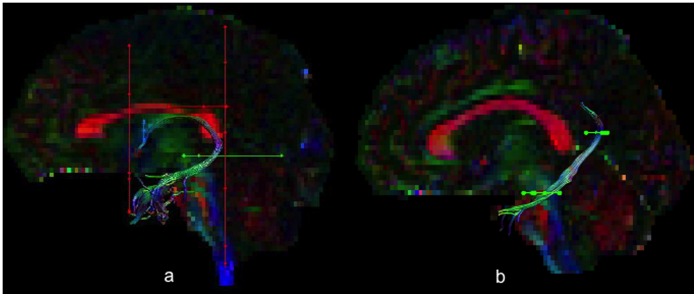
**Figure 1** illustrates the deterministic tractography reconstructions of a) the fornix and b) the parahippocampal cingulum on a parasagittal view of one individual’s colour coded diffusion map. Seed (OR) waypoint region of interest gates are illustrated in blue, AND gates in green and NOT gates in red.

A coronal OR ROI was placed around the anterior pillars and an axial AND ROI around the crus fornici. NOT ROIs were placed rostral to the anterior pillars, caudal to the crus fornici, around the corpus callosum and the upper pons (see Figure 1a).

#### Parahippocampal cingulum

One axial AND ROI was placed dorsal to the ventral limit of the splenium and another at the level of the pons-midbrain junction [17]. A NOT ROI was placed above the body of the corpus callosum caudal to the rostral-caudal midpoint of the body of the corpus callosum (see Figure 1b).

### Hippocampal and Whole Brain Volume Measures

Hippocampal volumes were generated with the *HippoQuant* software [33], developed by BioClinica SAS, Lyon, France. This is an adaptation of hippocampal outlining techniques (based on [34]) but involves placing approximately 80 landmark points, rather than tracing full outlines on each slice, with the remaining surface filled in by deforming a standard three-dimensional hippocampal template to match these landmark points. In a first step, T1-weighted images were displayed simultaneously in all three planes (axial, sagittal, coronal) and a trained rater placed multiple landmarks on the boundary of the hippocampus, paying particular attention to areas of difficult anatomical distinction (for example, the alveus and the boundary with the amygdala). The hippocampal measurements included the hippocampus proper (CA-1 to CA-4 sectors), dentate gyrus, alveus, fimbria and subiculum. Parahippocampal regions (entorhinal cortex and intervening white matter including perforant pathway) were excluded. The resulting hippocampal mask was further refined by a tissue-type segmentation step (i.e. voxels segmented as grey or white matter are included, CSF voxels excluded). The final hippocampal masks were then checked visually by the rater and edited for obvious errors. Hippocampal volumes were corrected for head size by calculating intracranial volume from summing segmented grey, white matter and CSF images (from the volumetric T1-weighted image) in Statistical Parametric Mapping 8 [35]. Brain parenchymal fraction (BPF), i.e. the volume of gray and white matter normalised to total intracranial volume to correct for head size, as a measure for whole brain volume was also obtained.

### Cognitive Assessment

All participants underwent neuropsychological assessment of working and episodic memory and executive function. Age-related changes for this sample were present only in episodic memory performance and were strongest for verbal free recall in the Free and Cued Selective Reminding task [36]. Performance in this task was also related to variation in fornix microstructure (FA) [24]. Changes in episodic memory in older age may, in some cases, be indicative of underlying neurodegenerative disease such as Alzheimer’s Disease and the prodromal manifestation of Mild Cognitive Impairment (MCI). MCI and Alzheimer’s disease are both associated with atrophy of the medial temporal lobe, including the hippocampus and fornix, and changes in BMI due to weight loss [37]. For these reasons, we employed verbal free recall performance in addition to verbal NART-IQ (see Table 1) as measures of individual variation in cognitive performance that would potentially confound any relationship between fornix microstructure and BMI, if covert Alzheimer’s neuropathology were driving these associations.

### Statistical Analyses

Statistical analyses were performed using Predictive Analytics Software (PASW) Statistics 18.0 [38]. BMI data were tested for the assumption of normal distribution with the Kolmogorov-Smirnov and Shapiro-Wilk tests. Two-tailed Pearson product moment correlations were calculated between BMI and white matter microstructural indices in the fornix and the parahippocampal cingulum, as well as with total hippocampal volume and brain parenchymal fraction. These correlations were carried out for all participants (n = 38) and only for individuals with BMI within normal-overweight range (n = 34) to ensure that correlations were not driven by a few extreme cases. Correlations between BMI and structural indices (10 in total) had to reach p<0.005 to comply with experiment-wise Bonferroni corrected significance level of 0.05.

Relationships were further explored separately for normal and overweight participants and females and males. The latter correlations were evaluated with one-tailed tests and were not further subjected to multiple-comparison corrections.

Pearson product moment correlations between white matter microstructural indices in the fornix and potentially confounding variables of age, education, NART-IQ, verbal free recall, systolic and diastolic blood pressure and BPF were calculated. Correlations (a total of 28 comparisons) had to reach p<0.002 to comply with experiment-wise Bonferroni corrected significance level of 0.05.

The role of potential confounds in the relationship between BMI and white matter microstructural indices were explored with partial correlations and multivariate hierarchical regression analyses, which were not further subjected to multiple comparison corrections. Multivariate hierarchical regression analysis was employed by entering all confounding variables first into a linear regression model with BMI as dependent variable, followed by the stepwise addition of the diffusion MRI indices in the fornix and the parahippocampal cingulum. Only one participant in the current study was diabetic – the exclusion of this individual did not alter any associations.

## Results

### Relationship between BMI and White Matter Microstructure

Both Kolmogorov-Smirnov and Shapiro-Wilk tests of normality produced non-significant results for the BMI data consistent with the null hypothesis of normality (F(38) = 0.08, p = 0.20; W(38) = 0.98, p = 0.66).

For all 38 participants there were positive correlations between BMI and fornix AD (r = 0.49; p≤0.002) and BMI and fornix MD (r = 0.46; p≤0.004) as well as a trend for a positive correlation between BMI and fornix RD (r = 0.37; p = 0.02). No correlations were observed between BMI and fornix FA (r = 0.08, p = 0.64) or any microstructural indices in the parahippocampal cingulum (FA: r = 0.20, p = 0.23; MD: r = −0.09, p = 0.61; RD: r = −0.14, p = 0.42; AD: r = 0.07, p = 0.68).

**Figure 2:**
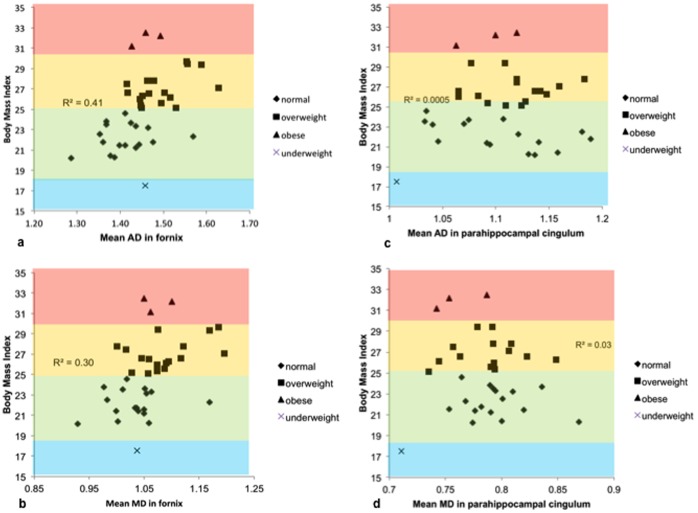
Scatterplots visualising the positive relationships between Body Mass Index (BMI) and a) average axial diffusivity (AD) (x10^−3^mm^2^.s^−1^) and b) mean diffusivity (MD) (x10^−3^mm^2^.s^−1^) in the fornix as well as the null relationships between BMI and c) AD and d) MD in the parahippocampal cingulum (collapsed across hemispheres) in a group of older adults (n = 38). R^2^ refers to the correlation coefficients based on the normal and overweight sample only (n = 34). BMI ranges are color coded with blue indicating underweight, green healthy, orange overweight and red obese ranges.

After the exclusion of the four extreme cases (three obese and one underweight individual) (n = 34) the positive correlations between BMI and axial and mean diffusivity in the fornix increased in magnitude (AD: r = 0.64, p≤0.001; MD: r = 0.55; p≤0.001) (see Table 2, Figure 2). A clearer trend for a relationship between BMI and fornix RD was observed (r = 0.45, p = 0.007), but again no correlations were found with fornix FA (r = 0.18, p = 0.32) or with any of the microstructural indices in the parahippocampal cingulum (FA: r = 0.13, p = 0.46; MD: r = 0.07, p = 0.69; AD: r = 0.14, p = 0.42; RD: r = 0.14, p = 0.42) (see Table 2, Figure 2).

**Table 2:** Relationship between Body Mass index and microstructural indices in the fornix and parahippocampal cingulum as well with whole brain and hippocampal volume for the normal and overweight individuals (BMI 20–30) (n = 34).

	BMI
HCV	0.26
BPF	0.12
Fornix FA	0.18
Fornix AD	**0.64** ***
Fornix RD	*0.45* **
Fornix MD	**0.55** ***
PHC FA	0.13
PHC AD	0.14
PHC MD	0.07
PHC RD	0.14

Abbreviations: AD: axial diffusivity; BMI: Body Mass Index; BPF: Brain Parenchymal Fraction; FA: Fractional Anisotropy; HCV: Hippocampal volume; MD: Mean diffusivity; PHC: parahippocampal cingulum (collapsed across hemispheres); RD: radial diffusivity. Pearson correlation coefficients significant at Bonferroni corrected level of p<0.005 are highlighted in bold and correlations with significance below the uncorrected 5% level are highlighted in italics.

** p≤0.01;

*** p≤0.001.

Further exploratory analyses (one-tailed) revealed that participants with normal weight (n = 17) showed no significant relationships between BMI and microstructural indices in the fornix (FA: r = 0.23, p = 0.19; MD: r = 0.12, p = 0.65; AD: r = 0.22, p = 0.20; RD: r = 0.01, p = 0.49). In contrast, overweight participants (n = 17) exhibited clear positive relationships (uncorrected) between all diffusivity measures in the fornix (MD: r = 0.44, p≤0.04; AD: r = 0.47, p≤0.03; RD: r = 0.42, p≤0.05) but not with fornix FA (r = −0.11, r = 0.34).

Females (n = 22) and males (n = 16) demonstrated comparable positive correlations between BMI and fornix AD (males: r = 0.45, p≤0.03; females: 0.46, p≤0.02) and fornix MD (males: r = 0.48, p≤0.03; females: 0.37, p≤0.04). Both groups exhibited comparable trends between BMI and fornix RD (males: r = 0.36, p = 0.09; females: r = 0.32, p = 0.07) but neither group showed any correlations between BMI and fornix FA (males: r = 0.08, p = 0.39; females: r = 0.12, p = 0.29).

### Relationships with Confounding Variables

Since the strongest correlations were found for the combined group of normal and overweight participants (BMI 18.5–30) and the aim of our study was to investigate factors that may contribute to an individual’s susceptibility to gain weight, the following analyses did not include the three obese and the one underweight individual (n = 34).

There were no correlations between BMI and any of the following variables: age (r = −0.04; p = 0.84), systolic and diastolic blood pressure (r = 0.01; p = 0.99 and r = −0.04; p = 0.84), education (r = −0.19; p = 0.28), NART-IQ (r = 0.28; p = 0.11), verbal free recall (r = 0.24; p = 0.17) or BPF (r = 0.17; p = 0.35).

Further, apart from the already reported positive relationships between fornix FA and age and verbal free recall respectively [24], there were no relationships between fornix microstructure and age, blood pressure, education, or BPF (see Table 3). However, trends (uncorrected for multiple comparisons) were observed between fornix diffusivity indices and NART-IQ as well as free recall (see Table 3).

**Table 3:** Relationship between microstructural indices in the fornix and demographic and cognitive variables.

Fornix	FA	MD	AD	RD
Age	**−0.66** ***	0.14	−0.14	0.31
BP systolic	−0.16	−0.06	−0.10	−0.01
BP diastolic	0.22	−0.13	−0.03	−0.20
Education	0.05	0.12	0.10	0.13
NART-IQ	−0.11	*0.37* *	*0.35* *	*0.36* *
Free recall	**0.56** ***	0.18	*0.43* *	−0.02
BPF	0.32	−0.13	0.03	−0.23

Abbreviations: AD: axial diffusivity; BP: Blood Pressure; BPF: Brain Parenchymal Fraction; FA: Fractional Anisotropy; Mean diffusivity; RD: radial diffusivity. Pearson correlation coefficients significant at Bonferroni corrected level of p<0.002 are highlighted in bold and correlations with significance below the 5% level (uncorrected) are highlighted in italics.

* p≤0.05;

*** p≤0.001 (as previously reported in [24]).

Partial correlations between BMI and fornix diffusivity indices (AD, MD, RD), accounting for the variation in potentially confounding variables of age, education, NART-IQ, free recall, blood pressure and brain volume did not remove, and in some cases increased, the magnitude of correlations. The partial correlations between BMI and fornix microstructural indices accounting for age were r = 0.64, p≤0.001 (AD); r = 0.56, p≤0.001 (MD); r = 0.49, p≤0.004 (RD); accounting for education: r = 0.68, p≤0.001 (AD); r = 0.58, p≤0.001 (MD); r = 0.50, p≤0.001 (RD); for NART-IQ: r = 0.59, p≤0.001 (AD); r = 0.52, p≤0.002 (MD); r = 0.44, p≤0.01 (RD); accounting for free recall: r = 0.62, p≤0.001 (AD); r = 0.53, p≤0.002 (MD); r = 0.46, p≤0.007 (RD); accounting for blood pressure (systolic and diastolic): r = 0.65, p≤0.001 (AD); r = 0.55, p≤0.001 (MD); r = 0.46, p≤0.01 (RD) and accounting for overall brain volume with BPF: r = 0.64, p≤0.001 (AD); r = 0.59, p≤0.001 (MD); r = 0.52, p≤0.002 (RD).

### Multivariate Analysis

Age, education, NART-IQ, verbal free recall, systolic and diastolic blood pressure and BPF were entered together into a hierarchical regression model with BMI as dependent variable, followed by the stepwise addition of all microstructural indices (AD, RD, MD, FA) in the fornix and the parahippocampal cingulum.

The adjusted *R^2^* for all confounding variables together (age, education, IQ, free recall, blood pressure, BPF) was 0.23 with no single variable making a significant independent contribution. The inclusion of white matter microstructural indices improved the fit of the model from 23% to 45% (adjusted *R^2^* = 0.45) with fornix AD being the only variable accounting for a significant proportion of the variance in BMI (t = 2.9, p≤0.01) independent of the other variables. The pattern of results was similar when free recall was not used as a covariate. In this case the inclusion of white matter microstructural indices improved the fit from 15% to 46% (adjusted *R^2^* = 0.46) with fornix AD the sole independent predictor (t = 3.5, p≤0.002).

### Relationship between Body Weight and Hippocampal Volume

There was no correlation between BMI and total hippocampal volume (r = 0.26; p = 0.14).

## Discussion

Positive relationships between BMI and fornix microstructure were observed in a group of older participants. These relationships were primarily driven by overweight but not obese individuals and were more pronounced after the exclusion of three obese and one underweight participant, so were not determined by outliers. The relationships between fornix microstructural indices and BMI were comparable between females and males, and were not confounded by age, education, IQ, episodic memory performance, high blood pressure, diabetes mellitus or inter-individual variation in whole brain volume. Thus, we propose that the observed correlation in our cognitively high functioning group of older people was not mediated by individual differences in demographic or intellectual variables, or by cardio-vascular risk states such as high blood pressure or diabetes.

The regulation of food intake is a complex process involving interactions between regions responsible for homeostatic, habitual, reward-related and mnemonic aspects of behavioural control. The hypothalamus monitors energy levels and responds to them adaptively through hunger and satiety signals, and is the hub for homeostatic aspects of food regulation. The hippocampus may contribute learning and memory processes that influence the control of appetitive and consummatory behavior, such as experiencing hunger at set meal times during the day [5], [39]. Other hippocampal interactions via the fornix are with the ventral striatum [11], a key structure in dopamine-based habitual reward mechanisms, ensuring positive reward associated with food intake. Medial prefrontal and orbitofrontal cortices contribute to food regulation by mediating higher level reward and cognitive control aspects [40], [41], important for the successful maintenance of a healthy body weight. In addition, a variety of subcortical sites innervate the hippocampus via the fornix [1].

All of these regions contribute unique attributes but need to interact with each other to ensure the adaptive and efficient regulation of food intake behaviour. For instance, hippocampal-prefrontal-ventral striatum circuits are involved in inhibitory aspects of response control [3] and also mediate the ability to delay anticipated reward [41]. Further, hippocampal-hypothalamic connections may facilitate learned patterns of food intake control and energy balance [39]. The fornix maintains projections between hippocampus and prefrontal cortex, ventral striatum, and hypothalamus respectively. Thus, one interpretation of the observed association between fornix microstructure and BMI is that the fornix, in connecting all of these regions, contributes critically to the interactive processes that underlie food intake regulation.

The relationship with BMI was specific to the fornix and was not observed for the parahippocampal cingulum. The parahippocampal cingulum also contains medial temporal lobe connections, but in contrast to the fornix is preferentially linked to occipital and parietal cortices, rather than the medial prefrontal cortex or hypothalamus [17]. Further within the current sample of normal and overweight adults, there was no correlation between BMI and hippocampal volume. Together, this pattern of results suggests that not all hippocampal/medial temporal lobe connections contribute substantially but only those that project directly to regions involved in food regulation such as the hypothalamus and the prefrontal cortex.

One other study [42] has reported a relationship between fornix microstructure and obesity. There are, however, a number of important differences in the present study. Stanek et al. [42] investigated white matter changes across the whole brain and found decreases in fornix FA (the authors did not report measures of diffusivity) in obese individuals. Obesity is associated with a number of metabolic and pathological abnormalities, so in this context structural brain changes are less surprising. In contrast, the present study observed changes in fornix diffusivity in individuals with BMI ranges in the normal and overweight range demonstrating that such effects are not exclusively related to obesity and perhaps emerge before the transition to obesity.

It should be noted that the absence of a correlation between variations in BMI and blood pressure and other health conditions may be due to range restrictions in the health variables and the known difficulties of quantifying lifetime risk exposures, especially when they are effectively controlled by medication, as in our sample. In addition, it is unlikely that biological features of depression would account for our results since participants were free of a history of major depressive disorder and – all but one individual, scored within normal range in the GDS15 with even that individual right on the cut-off score.

Alterations in fornix microstructure were specifically observed with axial, mean, and radial diffusivity but were not present for fractional anisotropy. Combined with our previous work on age-related changes in fornix microstructure and episodic memory in the same sample of older adults [24], we observed a double dissociation: partial volume corrected FA was significantly related with age and memory but not with BMI; whereas diffusivity indices were related to BMI but not to age or memory performance. This pattern of results suggests that FA and diffusivity indices may be sensitive to different microstructural aspects of white matter and may not always be interchangeable or directly comparable.

It is difficult to infer the precise biophysical alterations underlying changes in DTI based indices of FA, MD, AD and RD because these measures can be modulated by a number of parameters such as myelination, axonal density and diameter and the complexity of the underlying fibre architecture [43]. Interpretations of DTI based diffusivity indices in terms of axonal loss or myelin alterations are primarily based on results from animal studies that have investigated single coherently aligned fibres [44]. Since the fibre architecture in the human brain, however, is complex and scenarios such as crossing fibres affect the magnitude of DTI based indices (FA for instance drops in areas of crossing fibres), interpretations based on single fibre experiments may not be appropriate. Our results suggest that variations in body composition relate to changes in fornix microstructure. However, in order to make inferences about the biophysical basis of these changes more direct microstructural indices of, for instance, axonal diameter [45] or density [46] will have to be employed in future studies.

The present study reports a correlational result and thus cannot infer direction of potential causality. Neurodevelopmental and inherited factors might predispose an individual to less well developed fornix connections and, hence, to less efficient communication and control within food regulatory circuits, leading to increased food intake. On the other hand, it may also be possible that body fat itself has detrimental effects on fornix white matter leading to increased vulnerability to pathological mechanisms associated, for instance, much later with exaggerated dementia risk [47]. If this account is correct, then these effects must be anatomically specific given the lack of correlations in the parahippocampal cingulum.

The question of directionality as well as the functional significance of our result and the role of other potential factors such as hormonal modulation (e.g. cortisol) [48] need to be addressed in future prospective studies. In the present context, we might expect BMI associated changes in fornix AD to relate to the neurophysiological and psychological correlates of food intake control such as differential responding to food cues in satiety or food restricted states that could be studied with functional neuroimaging.

In summary, this is the first study to demonstrate a robust relationship between microstructural white matter indices in the fornix tract and variation in BMI within normal and overweight range in a group of older adults. These results have important implications for the understanding of neural factors contributing to an individual’s susceptibility to weight gain.
